# Supplementary material for: C-Reactive Protein and Neutrophil/Lymphocytes Ratio: Prognostic Indicator for Doubling Overall Survival Prediction in Pancreatic Cancer Patients
Source: J Clin Med. 2019 Oct 25;8(11):1791. doi: 10.3390/jcm8111791 (PMC6912559; doi:10.3390/jcm8111791)
Supplement: Supplementary file 1 [file jcm-08-01791-s001.pdf]

Supplemental Table

Table S1. List of all applied chemotherapy agents, regardless of therapy line.

FOLFIRINOX
Gemcitabine and Nab-paclitaxel
Nanoliposomal Irinotecan
Nab-paclitaxel
Gemcitabine- Erlotinib
Gemcitabine- Oxaliplatin
Docetaxel
Doxorubicin

Supplemental Graphic

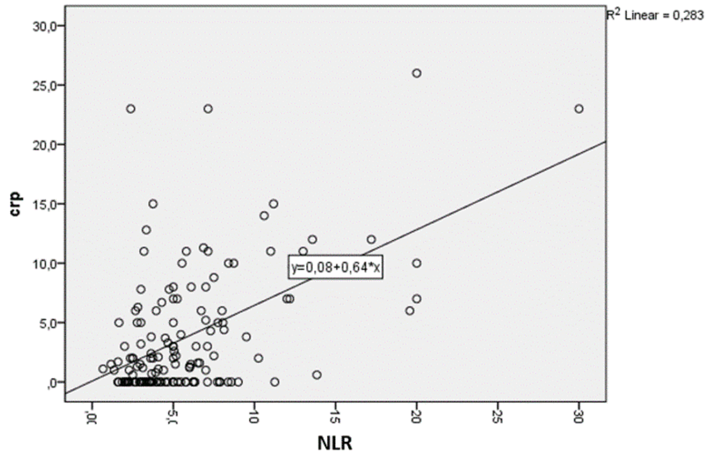

Figure S1 supplemenatal: We found a weak ($R^2 = 0.25$) but significant association between CRP and NLR elevation ($p < 0.001$).
